# Massive tarsal coalition with extended tarsometatarsal coalition in a child: a case report

**DOI:** 10.3389/fped.2024.1362142

**Published:** 2024-09-11

**Authors:** Lei Yang, Xiaodong Yang, Jun Jiang, Xueyang Tang

**Affiliations:** West China Hospital, Sichuan University, Chengdu, China

**Keywords:** tarsal coalition, foot deformity, synostosis, pediatric, case report

## Abstract

Tarsal coalition refers to the union of two or more tarsal bones, which mostly involves the calcaneonavicular and talocalcaneal joints; it is rarely found in multiple unions or unions extended to the metatarsal bones. Nearly 50% of cases occur bilaterally and can be either symmetrical or asymmetrical. We described a rare case of symmetrically bilateral tarsal coalitions involving all the tarsal bones, except for the medial cuneiform, and extending to the fourth metatarsal bones in a 5-year-old boy. This patient exhibited no obvious symptoms and had not received any further intervention, only regular follow-up. To our knowledge, this is the first report of this type of massive coalition involving the union of six tarsals and one tarsometatarsal bilaterally.

## Introduction

The tarsus connects the lower leg to the metatarsals and includes separate bones such as calcaneus, talus, navicular, cuboid, and cuneiform bones (1). A tarsal coalition refers to a union between two or more of these bones, which can be connected by fibrous, cartilaginous, or osseous tissue (2). The true incidence of tarsal coalition remains unclear due to the rarity and asymptomatic nature of this disease, which has been reported as 1%–2% in most previous studies (3, 4). The coalition mostly occurs in the calcaneonavicular or the talocalcaneal joint, which accounts for nearly 90% of all cases (5, 6). Coalitions involving more than three joints or other tarsal bones are less frequent, and massive tarsal coalitions extending to the metatarsals are even rarer (7, 8). In this report, we describe a rare case of coalitions involving six tarsal bones (calcaneus, talus, navicular, cuboid, intermediate, and lateral cuneiform bones) and the fourth metatarsal bones bilaterally.

## Case report

A 5-year-old boy was referred to the pediatric orthopedic clinic of our hospital by his parents due to a mild deformity in both feet. The abnormal foot shape had not been noted by his parents until half a year ago, and there were no complaints of foot pain or other symptoms. No pain or deformity was noted in other joints, the spine, or any other parts of his body. There was no family history of a similar condition, nor was there any history of trauma to his feet. Also, there was no history of neuromuscular, infectious, or syndromic disease.

On general examination, no spinal anomalies or limited range of motion in other joints were found. There was only a mild degree of forefoot abduction and lateral foot widening from the appearance of feet, with an acceptable medial longitudinal arch, normal standing posture, and gait. There was no appreciable subtalar or hindfoot motion in both his feet. Palpation revealed no swelling or tenderness on either side, and there was no tightness in the Achilles tendons. There was no restriction in ankle dorsiflexion, inversion, and eversion bilaterally.

**Figure 1:**
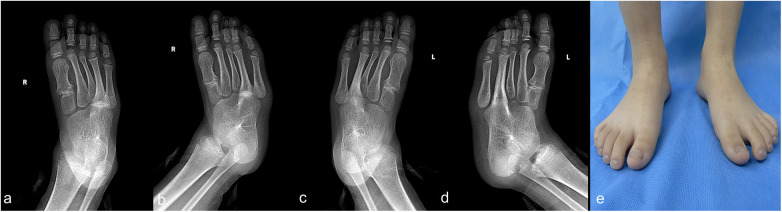
Images of a 5-year-old boy with bilateral massive tarsal coalitions. The anterior–posterior and oblique views of the right foot **(a**,**b)** demonstrate a massive coalition involving the calcaneus, talus, navicular, cuboid, intermediate cuneiform, lateral cuneiform, and the fourth metatarsal bones. The anterior–posterior and oblique views of the left foot **(c**,**d)** demonstrate a massive coalition involving the calcaneus, talus, navicular, cuboid, intermediate cuneiform, lateral cuneiform, and the fourth metatarsal bones. The cosmetic image of both feet **(e)**.

**Figure 2:**
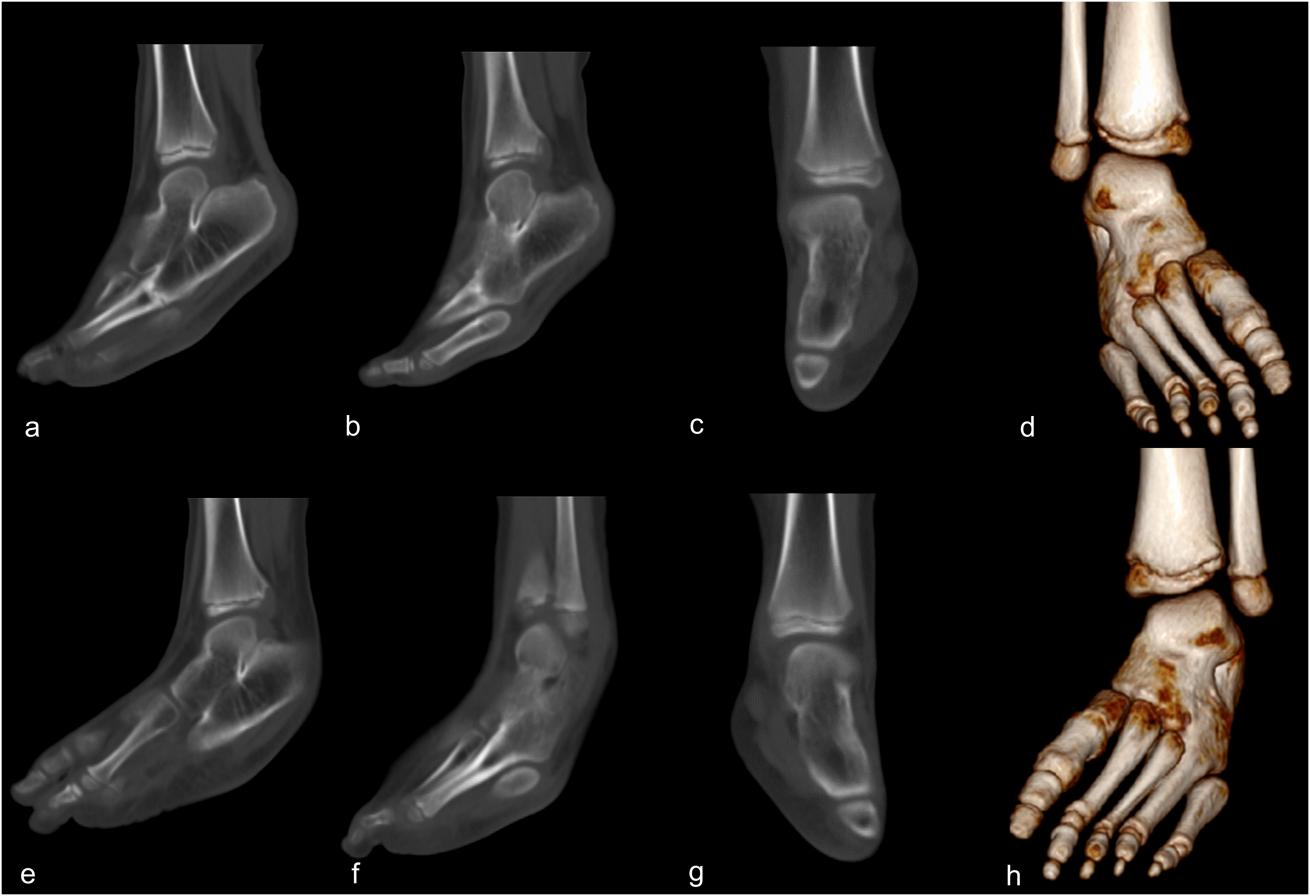
Computed tomography scan of a 5-year-old boy with bilateral massive tarsal coalitions. The sagittal views of the right foot show the osseous unions of the tarsal bones **(a)** and the union extending to the fourth metatarsal bone **(b)** The coronal **(c)** and 3D reconstructed **(d)** images of the right foot. The sagittal views of the left foot show the osseous unions of the tarsal bones **(e)** and the union extending to the fourth metatarsal bone **(f)**. The coronal **(g)** and 3D reconstructed **(h)** images of the left foot.

Laboratory test results were within normal ranges. Plain radiographs revealed bilateral, symmetric, and multiple coalitions involving the calcaneus, talus, navicular, cuboid, intermediate, and lateral cuneiform bones as well as the fourth metatarsals (Figure 1). Computed tomography confirmed the osseous unions of all these coalitions (Figure 2).

Considering that the patient was young, with an immature skeleton, and no obvious symptoms, no further intervention was carried out. We recommended a regular follow-up at 1-year intervals until the patient attains physical maturity or becomes symptomatic. Over the last 1 year of observation, the patient has remained asymptomatic, and the condition has not progressed.

## Discussion

The reported incidence of tarsal coalitions in the literature varies widely, ranging from 1% to 12.7% (3, 4, 9). The true frequency in the general population remains uncertain, and some authors believe it is underestimated because most patients are asymptomatic or present with atypical symptoms (10, 11). Flatfoot, pain, abnormally shaped feet, repeated ankle sprains, and loss of mobility are reported as the clinical signs suggestive of tarsal coalitions in childhood; however, none of these signs are diagnostic (2, 12, 13). In addition, many patients with tarsal coalitions might not present with symptoms until they are older than 12 years, because of the progressive ossification of the coalition during this period. Thus, tarsal coalition in young children is difficult to detect, especially in those under the age of 6, and the true incidence of tarsal coalition is not easy to determine (2, 14, 15).

Most coalitions involve just two tarsals, commonly the calcaneus, navicular, or talus, with nearly 50% of the cases being bilateral (3, 16). Multiple or massive tarsal coalitions are less frequent and account for less than 10% of all the cases, among which some are associated with hereditary syndromes (17, 18). In addition, most massive coalitions mainly involve the tarsals, and the type that extends to the metatarsal is even less prevalent. Thus, the non-syndromic, bilateral, massive tarsal coalitions involving metatarsals are extremely rare. Alatassi et al. (14) reported a similar case of bilateral non-syndromic massive tarsal and tarsometatarsal coalitions in a 4-year-old girl who presented only with the abnormal shape of her feet. There was no foot pain, abnormal gait, or other symptoms. Compared to the case described in our study, the coalition described by Alatassi et al. was asymmetrical and more complex, involving all the tarsals and the second, third, and fourth metatarsals on the right foot and all the tarsals and the third metatarsal on the left foot.

Yeganeh et al. (18) also reported a rare case of bilateral massive coalition in a 24-year-old man, where all the tarsal and tarsometatarsal bones were fused into one integrated tarsal. The main concern of the patient was mild ankle pain, which might result from secondary osteoarthritis, which responded well to conservative treatment with oral ibuprofen. Compared to the case described in our study, the patient reported by Yeganeh et al. experienced typical pain in the ankle and had a more extensive fusion involving all tarsal and tarsometatarsal bones.

There is no evidence to support any therapeutic intervention for asymptomatic tarsal coalitions, as most simple asymptomatic tarsal coalitions do not lead to further problems (14, 19). However, regular follow-up is necessary for massive coalitions without symptoms, as some patients may experience symptoms such as limited joint motion or secondary osteoarthritis in the future (14, 17, 18).

For symptomatic patients, a conservative protocol is recommended as the initial treatment, including non-steroidal anti-inflammatory drugs, changing habits, and the use of orthosis or cast immobilization (4, 19, 20). If there is no response to symptoms after conservative therapy, surgical treatment may be considered (21). Surgical resection of the coalition, with or without interposition in the resection gap, has been widely reported as an effective method for treating patients with persistent symptoms (22). If resection treatment fails, selective or triple joint arthrodesis surgery is recommended as a salvage procedure (4). For patients with a massive coalition, resection treatment may not be applicable. The conservative protocol or osteotomy procedure to reshape the foot deformity may be more appropriate. However, due to the rarity of cases, there is still a lack of evidence regarding the treatment for massive coalitions. In addition, for skeletally immature children, there may be an alteration of the condition and treatment as the child grows.

## Conclusions

A massive tarsal coalition is extremely rare and usually presents asymptomatically in childhood. We described a rare case of a young child with massive coalitions involving six tarsal bones and one metatarsal bilaterally. Conservative treatment and regular follow-up are the initial choices for most patients with massive coalitions.

## Data Availability

The original contributions presented in the study are included in the article/Supplementary Material, further inquiries can be directed to the corresponding author.
